# Novel Mechanism for Disrupted Circadian Blood Pressure Rhythm in a Rat Model of Metabolic Syndrome—The Critical Role of Angiotensin II

**DOI:** 10.1161/JAHA.113.000035

**Published:** 2013-06-21

**Authors:** Daisuke Sueta, Keiichiro Kataoka, Nobutaka Koibuchi, Kensuke Toyama, Ken Uekawa, Tetsuji Katayama, Ma MingJie, Takashi Nakagawa, Hidefumi Waki, Masanobu Maeda, Osamu Yasuda, Kunihiko Matsui, Hisao Ogawa, Shokei Kim‐Mitsuyama

**Affiliations:** 1 Department of Pharmacology and Molecular Therapeutics, Kumamoto University Graduate School of Medical Sciences, Kumamoto, Japan (D.S., K.K., N.K., K.T., K.U., T.K., M.M.J., T.N., S.K.M.); 2 Department of Physiology, Wakayama Medical University School of Medicine, Wakayama, Japan (H.W., M.M.); 3 Department of Cardiovascular Clinical and Translational Research, Kumamoto University Hospital, Kumamoto, Japan (O.Y.); 4 Department of General Medicine, Yamaguchi University Hospital, Yamaguchi, Japan (K.M.); 5 Department of Cardiovascular Medicine, Kumamoto University Graduate School of Medical Sciences, Kumamoto, Japan (H.O.)

**Keywords:** angiotensin, circadian rhythm, hypertension, nervous system, obesity, sympathetic

## Abstract

**Background:**

This study was performed to determine the characteristics and mechanism of hypertension in SHR/NDmcr‐cp(+/+) rats (SHRcp), a new model of metabolic syndrome, with a focus on the autonomic nervous system, aldosterone, and angiotensin II.

**Methods and Results:**

We measured arterial blood pressure (BP) in SHRcp by radiotelemetry combined with spectral analysis using a fast Fourier transformation algorithm and examined the effect of azilsartan, an AT1 receptor blocker. Compared with control Wistar‐Kyoto rats (WKY) and SHR, SHRcp exhibited a nondipper‐type hypertension and displayed increased urinary norepinephrine excretion and increased urinary and plasma aldosterone levels. Compared with WKY and SHR, SHRcp were characterized by an increase in the low‐frequency power (LF) of systolic BP and a decrease in spontaneous baroreflex gain (sBRG), indicating autonomic dysfunction. Thus, SHRcp are regarded as a useful model of human hypertension with metabolic syndrome. Oral administration of azilsartan once daily persistently lowered BP during the light period (inactive phase) and the dark period (active phase) in SHRcp more than in WKY and SHR. Thus, angiotensin II seems to be involved in the mechanism of disrupted diurnal BP rhythm in SHRcp. Azilsartan significantly reduced urinary norepinephrine and aldosterone excretion and significantly increased urinary sodium excretion in SHRcp. Furthermore, azilsartan significantly reduced LF of systolic BP and significantly increased sBRG in SHRcp.

**Conclusions:**

These results strongly suggest that impairment of autonomic function and increased aldosterone in SHRcp mediate the effect of angiotensin II on circadian blood pressure rhythms.

## Introduction

Metabolic syndrome (MetS) is associated with an increase in cardiovascular morbidity and mortality. A large proportion of hypertensive patients with MetS are characterized by disrupted circadian blood pressure (BP) rhythms such as nocturnal nondipper‐type, riser‐type, or morning surge–type hypertension.^1–2^ Such disrupted diurnal BP rhythms are associated with a high rate of cardiovascular events.^3–8^ However, the precise mechanism of how circadian BP rhythms become disrupted in MetS remains to be defined.

SHR/NDmcr‐cp(+/+) rats (SHRcp) are a derivative of spontaneously hypertensive rats (SHR) and have a spontaneous nonsense mutation in the leptin receptor gene^9^. They manifest hypertension, obesity, insulin resistance, glucose intolerance, and dyslipidemia.^10–12^ Thus, SHRcp are regarded as a useful rat model of MetS. Previous studies^10–11^ have shown that SHRcp exhibit an increase in plasma aldosterone levels despite no increase in plasma renin activity and marked nephropathy, which is at least partially attributed to mineralocorticoid receptor activation. However, the characteristics and mechanism of hypertension in SHRcp are still unknown.

To test our hypothesis that SHRcp may have different characteristics and mechanisms of hypertension from SHR, we examined the circadian BP rhythm, the autonomic nervous system and the effect of azilsartan,^13–17^ a new AT1 receptor blocker (ARB), in SHRcp and compared them with SHR and Wistar‐Kyoto rats (WKY). We obtained the first evidence that SHRcp exhibit a nondipper‐type hypertension. This impaired diurnal BP variation in SHRcp was attributed to AT1‐receptor‐mediated autonomic dysfunction and aldosterone.

## Methods

### Experimental Animals

All procedures were performed in accordance with institutional guidelines for animal research and approved by the Animal Care and Use Committee of Kumamoto University.

Male WKY, male SHRcp, a rat model of metabolic syndrome, and male SHR were purchased from Japan SLC (Shizuoka, Japan). All rats were housed in an animal facility with a 12‐hour light–dark cycle and were given standard chow and water ad libitum.

### Oral Dosing of Azilsartan in SHRcp, SHR, and WKY

Azilsartan was kindly supplied by Takeda Pharma Co Ltd. (Tokyo, Japan). Azilsartan was suspended in 0.5% methylcellulose and administered orally to SHRcp, SHR, and WKY by gastric gavage at the beginning of the dark period once daily.

### Experiment I: Comparison Among SHR/NDmcr‐cp (SHRcp), SHR, and WKY

Miniaturized telemetry devices were implanted into 9‐week‐old SHRcp, age‐matched SHR, and age‐matched WKY (as described in detail below). Blood pressure (BP) and heart rate (HR) variability, low‐frequency power (LF) of systolic BP, and spontaneous baroreceptor reflex gain (sBRG) were monitored during the dark and light periods.

SHRcp, age‐matched SHR, and age‐matched WKY were housed in metabolic cages from 10 to 14 weeks of age, and a 12‐hour urine sample in each light and dark period was collected from each rat to compare urinary norepinephrine, aldosterone, and electrolyte excretion among the 3 strains.

### Experiment II: Effect of 0.1, 0.3, and 1.0 mg/kg per Day of Once‐Daily Dosing of Azilsartan on SHRcp, SHR, and WKY

Nine‐week‐old SHRcp, SHR, and WKY with surgically implanted telemetry devices were allowed a recovery period of 2 weeks. Baseline BP and HR measurements were recorded for 7 days. After that, SHRcp, SHR, and WKY were orally administered azilsartan for 3 weeks (0.1 mg/kg per day for the first week, 0.3 mg/kg per day for the second week, and 1 mg/kg per day for the third week) to examine the effect of azilsartan on diurnal BP and HR rhythms.

Furthermore, in separate experiments, the effect of azilsartan on urinary norepinephrine, aldosterone, and electrolyte excretion in SHRcp, SHR, and WKY was compared with vehicle treatment (0.5% methylcellulose). After 3 weeks of oral dosing of azilsartan, all rats were anesthetized with isoflurane, arterial blood was immediately collected by cardiac puncture, and serum was collected by centrifugation and stored at −80°C until use.

### Experiment III: Effect of Azilsartan on Vasomotor Sympathetic Tone and Baroreceptor Reflex Function in SHRcp

Nine‐week‐old SHRcp with surgically implanted telemetry devices were allowed 2 weeks of recovery.After recording baseline BP and HR measurements for 1 week, SHRcp were orally given 1 mg/kg of azilsartan or vehicle (0.5% methylcellulose) at 8 pm once daily for 4 weeks to examine the effect of azilsartan on BP variability, HR variability, LF of systolic BP, and sBRG.

### Experiment IV: Effect of Short‐Term Azilsartan Administration on Urinary Sodium Excretion in SHRcp

Eleven‐week‐old SHRcp were housed in metabolic cages to examine the effect of short‐term azilsartan treatment on urinary sodium excretion. The SHRcp were acclimatized to the metabolic cages for 48 hours (2 days), then 24‐hour urine was collected with metabolic cages to measure urinary sodium excretion per day on the third and fourth days before azilsartan treatment, and finally 1 mg/kg of azilsartan or vehicle (0.5% methylcellulose) was orally given once daily for 7 days to SHRcp in the metabolic cages.

### Measurement of Arterial BP Using Radiotelemetry

We used a telemetry system (Data Sciences International, St. Paul, MN) to record arterial pressure in SHRcp, SHR, and WKY, as described in detail previously.^18–20^ The validity of our method has been well established.^18–20^ The system consisted of 3 basic elements: (1) a transmitter for monitoring arterial pressure (TA11PA‐C40; Data Sciences International, St. Paul, MN), (2) a receiver (RPC‐1), and (3) an adapter (R11CPA) with an ambient pressure monitor (APR‐1) to output analog signals of arterial pressure. The system is calibrated relative to atmospheric pressure. A computer‐based data acquisition system was used to acquire, display, store, and analyze the data.

The transmitter was implanted 14 days before starting the telemetry recordings. Each rat was anesthetized with isoflurane, a midline incision in the abdominal wall was made with the rat in a supine position, and the tip of the catheter of the transmitter was inserted into the abdominal aorta. The transmitter was sutured to the ventral wall of the abdominal cavity. BP and HR data were obtained from the animal and recorded using a computer system (DATAQUEST ART4.2 Acquisition; Data Sciences International, St. Paul, MN). The data were recorded with 30‐second averages every 5 minutes for BP, HR, and locomotor activity and with 5‐minute averages every 60 minutes for LF of systolic BP and sBRG. Baseline BP and HR measurements were recorded for 7 days before the drug treatment.

### Evaluation of Vasomotor Sympathetic Tone and Spontaneous Baroreceptor Reflex Function

The magnitude of power was integrated in both the low‐frequency (LF) band between 0.27 and 0.75 Hz and the high‐frequency (HF) band (0.75 to 3.3 Hz).^21^ In our previous report, we validated that the LF power in the spectral density of systolic arterial pressure variability reflects vasomotor sympathetic tone.^18–20^ To evaluate time‐dependent changes in baroreceptor reflex function, baroreceptor reflex gain was determined from spontaneous changes in systolic BP and pulse interval using a modified time‐series method,^22^ as described previously.^18–20^ We only used positive slope values to avoid contaminating our baroreceptor reflex data with nonbaroreceptor‐mediated changes in the pulse interval.

### Measurement of Urinary and Plasma Variables

Blood biochemistry measurements were performed at SRL Inc (Tokyo, Japan). Urine aldosterone was measured with a kit (Cayman Chemical Company, Ann Arbor, MI). Other urine biochemistry measurements were performed at SRL Inc (Tokyo, Japan).

### Statistical Analysis

The method of statistical analysis used in each experiment is described in all figure legends. All data are presented as means±SEMs. Normality was tested with the Shapiro–Wilk test, and Bartlett's test was performed to examine whether variances were similar across comparison groups. When data were normally distributed and variances were similar across comparison groups, the statistical significance of differences was assessed by analysis of variance (ANOVA). Except for the data on effect of short‐term azilsartan treatment on urinary sodium excretion, statistical significance was determined with 2‐ or 3‐factor ANOVA (with repeated measures where appropriate) to evaluate the main and interactive effects of strains, periods, weeks, or azilsartan followed by Tukey's test for multiple comparisons, using SAS 9.1.3 (SAS Institute Inc, Cary, NC). In the case of 3‐factor ANOVA, individual pairwise comparisons were made with an unpaired *t* test with Bonferroni's correction. The data on the effect of short‐term azilsartan treatment on urinary sodium excretion was analyzed with 1‐factor ANOVA with repeated measures followed by post hoc Bonferroni's multiple comparisons test. Data were analyzed with Kruskal–Wallis test followed by post hoc Steel–Dwass's multiple comparison test when a normal distribution was not confirmed or similar variances were not obtained among comparison groups. In all tests, differences were considered statistically significant at *P*<0.05.

## Results

### Circadian Rhythms of Locomotor Activity, Blood Pressure, and Heart Rate in SHRcp, SHR, and WKY

**Figure 1.**
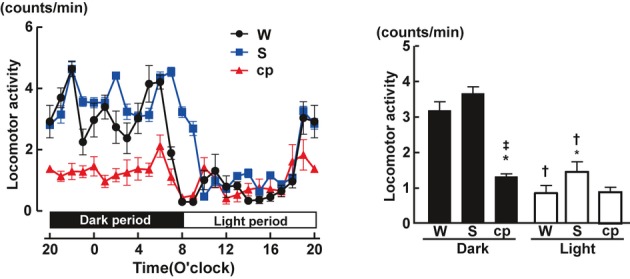
Circadian rhythms of locomotor activity revealed by 24‐hour recording. Left panel indicates hourly recordings of locomotor activity during 12‐hour dark and 12‐hour light periods. Right panel shows 12‐hour average locomotor activity during dark and light periods. Dark, 12‐hour dark period (20:00 to 8:00); light, 12‐hour light period (8:00 to 20:00). Values are the mean±SEM (n=5 in each group). Statistical analysis was performed by 2‐factor ANOVA followed by a post hoc Tukey's test. Locomotor activity was significantly influenced by strain (*P*<0.01), and period (*P*<0.01). *P<0.01 vs W within the same period; ‡*P*<0.01 vs S within the same period; †*P*<0.01 vs the same strain in the dark period. W indicates Wistar–Kyoto rats; S, SHR rats; cp, SHR/NDmcr‐cp(+/+) rats; SEM, standard error of the mean; ANOVA, analysis of variance.

**Figure 2.**
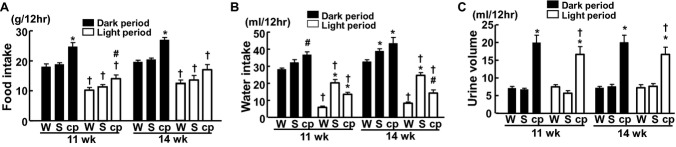
Food intake (A), water intake (B), and urine volume (C) in 11‐ and 14‐week‐old WKY, SHR, and SHRcp during dark and light periods. Values are the mean±SEM (n=6 in each group). Statistical analysis was performed by 2‐factor ANOVA with repeated measures followed by a post hoc Tukey's test. Food intake was significantly influenced by strain (*P*<0.01) and period (*P*<0.01). Water intake was significantly influenced by strain (*P*<0.01) and period (*P*<0.01). Urine volume was significantly influenced by strain (*P*<0.01), but not influenced by period (*P*=0.4375). #*P*<0.05; *P<0.01 vs W within the same period; †*P*<0.01 vs the same strain in the dark period. WKY and W indicate Wistar–Kyoto rats; S, SHR rats; SHRcp and cp, SHR/NDmcr‐cp(+/+) rats; SEM, standard error of the mean; ANOVA, analysis of variance.

As shown in Figure 1, locomotor activity of WKY and SHR was significantly greater during the dark, or active, period than during the light, or inactive, period (*P*<0.01). Locomotor activity of SHRcp during the dark period was significantly less than that of WKY (*P*<0.01) and SHR (*P*<0.01). There was no significant difference in locomotor activity between WKY and SHRcp during the light period, whereas locomotor activity of SHR during light period was larger than that of WKY during the same period (*P*<0.05). As shown in Figure 2, food intake, water intake, and urine volume in SHRcp were significantly greater than those in WKY during the dark and light periods at both 11 and 14 weeks of age.

**Figure 3.**
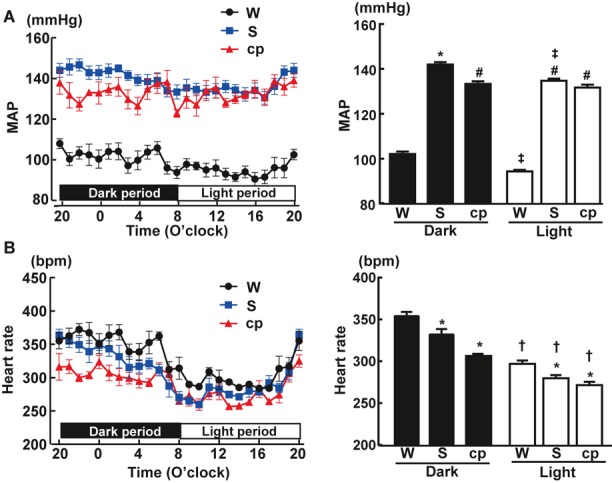
Circadian rhythms of mean arterial pressure (A) and heart rate (B) revealed by 24‐hour recording. The left panel in (A) shows hourly recordings of mean arterial pressure (MAP) over 24 hours, and the right panel in (A) indicate the average MAP during the 12‐hour dark and 12‐hour light periods. The left panel in (B) shows hourly recordings of heart rate over 24 hours, and the right panel in (B) indicate the average heart rate during 12‐hour dark and 12‐hour light periods. Values are expressed as the mean±SEM (n=5 in each group). Statistical analysis was performed by Kruskal–Wallis test followed by post hoc Steel–Dwass's multiple comparison test (A) and 2‐factor ANOVA followed by post hoc Tukey's test (B). MAP was significantly influenced by strain (*P*<0.01) and period (*P*<0.01). Heart rate was significantly influenced by strain (*P*<0.01) and period (*P*<0.01). #*P*<0.05; *P<0.01 vs W within the same period; ‡*P*<0.05; †*P*<0.01 vs the same strain in the dark period. MAP indicates mean arterial pressure; W, Wistar–Kyoto rats; S, SHR rats; cp, SHR/NDmcr‐cp(+/+) rats; SEM, standard error of the mean; ANOVA, analysis of variance.

As shown in Figure 3A, mean arterial pressure (MAP) in WKY was significantly lower during the light period than during the dark period (94.4±0.7 versus 102.1±1.0 mm Hg; *P*<0.05), indicating that WKY showed dipper‐type BP. MAP in SHR was also significantly lower during the light period than during the dark period (134.8±0.9 versus 142.0±1.1 mm Hg; *P*<0.05). Thus, SHR displayed dipper‐type hypertension. On the other hand, MAP in SHRcp did not differ between the dark and light periods (133.4±1.0 versus 131.7±1.3 mm Hg), indicating that SHRcp displayed nondipper‐type hypertension.

As shown in Figure 3B, HR was significantly lower during the light period than during the dark period in WKY (*P*<0.01), SHR (*P*<0.01), and SHRcp (*P*<0.01) rats. HR was slightly lower in SHRcp and SHR than in WKY over 24 hours.

### Effect of Azilsartan on Body Weight in SHRcp, SHR, and WKY

**Figure 4.**
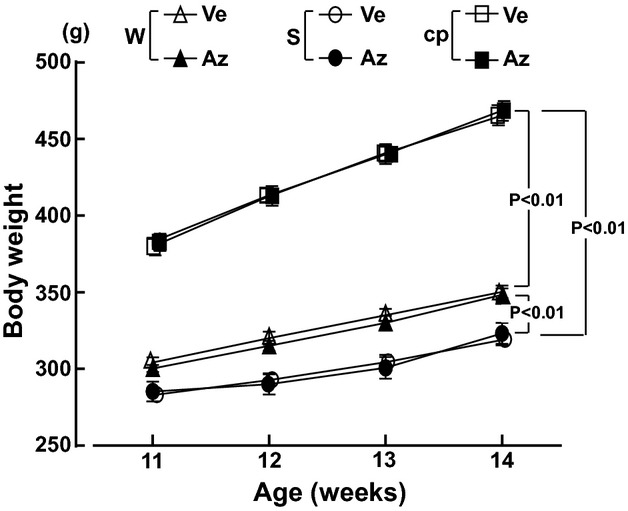
Effects of azilsartan on body weight in WKY, SHR, and SHRcp. Values are expressed as the mean±SEM (n=6 in each group). Statistical analysis was performed by 2‐factor ANOVA with repeated measures followed by post‐hoc Tukey's test. Body weight was significantly influenced by strain (*P*<0.01), but not influenced by azilsartan (*P*=0.8928). WKY and W indicate Wistar–Kyoto rats; S, SHR rats; SHRcp and cp, SHR/NDmcr‐cp(+/+) rats; SEM, standard error of the mean; ANOVA, analysis of variance; Ve, vehicle treatment; Az, azilsartan treatment.

Body weight of SHRcp was much greater than that of SHR (*P*<0.01) or WKY (*P*<0.01). Compared with vehicle treatment, azilsartan treatment did not significantly affect body weight of SHRcp, SHR, or WKY throughout the treatment (Figure 4).

### Effect of Azilsartan on Blood Pressure in SHRcp, SHR, and WKY

**Figure 5.**
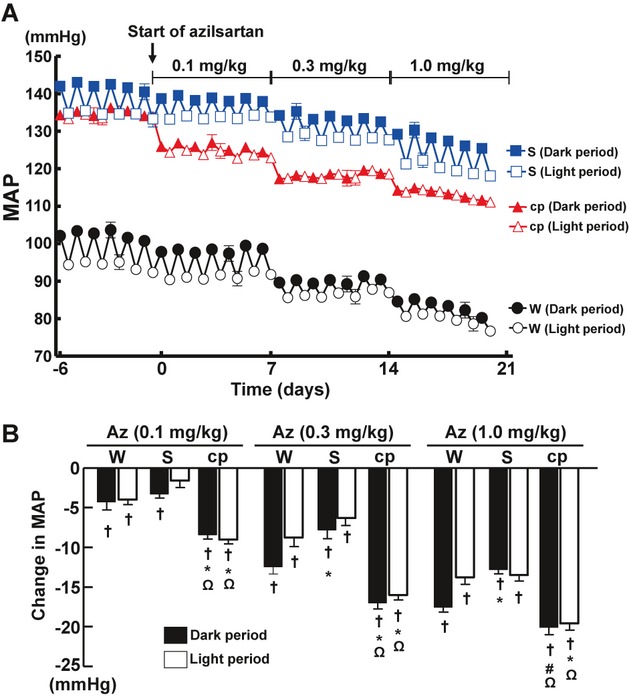
Effects of azilsartan on mean arterial pressure (MAP) in WKY, SHR, and SHRcp during dark and light periods. A, 12‐Hour average MAP in WKY, SHR, and SHRcp during dark and light periods. Azilsartan at 0.1 mg/kg for 7 days, 0.3 mg/kg for 7 days, and then 1.0 mg/kg for 7 days was given to WKY, SHR, and SHRcp by gastric gavage at the beginning of the dark period (20:00) once a day. B, Average change in 12‐hour MAP in WKY, SHR, and SHRcp during dark and light periods after 7 days of dosing of azilsartan at each dose (0.1, 0.3, and 1.0 mg/kg).. Values are the mean±SEM (n=5 in each group). Statistical analysis was performed by 3‐factor ANOVA with repeated measures followed by a post hoc Tukey's test. Individual pairwise comparisons were made with the unpaired *t* test with Bonferroni's correction after ANOVA analysis. Change in MAP was significantly influenced by strain (*P*<0.01), period (*P*<0.05), and dose of azilsartan (*P*<0.01). †*P*<0.01 vs the same strain within the same period before azilsartan treatment; #*P*<0.05; *P<0.01 vs W within the same dose and the same period; Ω*P*<0.01 vs SHR within the same dose and the same period. MAP indicates mean arterial pressure; WKY and W, Wistar–Kyoto rats; S, SHR rats; SHRcp and cp, SHR/NDmcr‐cp(+/+) rats; SEM, standard error of the mean; ANOVA, analysis of variance; Az, azilsartan treatment.

As shown in Figure 5, once‐daily oral dosing of azilsartan caused a dose‐dependent decrease in BP in SHRcp, SHR, and WKY. However, blood pressure reduction by azilsartan treatment significantly differed among the 3 strains. As shown by the change in MAP by each dose of azilsartan treatment in Figure 5B, the blood pressure–lowering effect of azilsartan at all doses (0.1, 0.3, and 1.0 mg/kg) was significantly greater in SHRcp than in SHR and WKY.

The above doses of azilsartan did not significantly affect locomotor activity, food intake, water intake, or urine volume in SHRcp, SHR, and WKY (data not shown).

### Effect of Azilsartan on Urinary Norepinephrine and Aldosterone Excretion, Serum Aldosterone, and Urinary Sodium Excretion in SHRcp, SHR, and WKY

**Figure 6.**
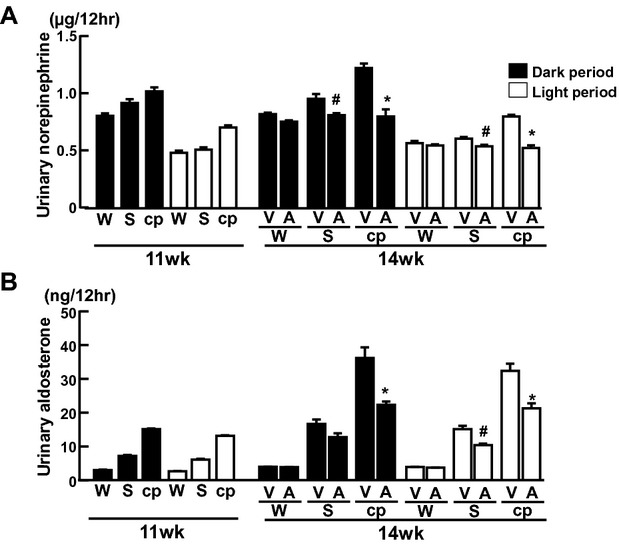
Effects of azilsartan on 12‐hour urinary excretion of norepinephrine (A) and aldosterone (B) during dark and light periods in WKY, SHR, and SHRcp. Values are the mean±SEM (n=12 in each group of 11‐week‐old rats, n=6 in each group of 14‐week‐old rats). Statistical analysis was performed by 3‐factor ANOVA with repeated measures followed by a post hoc Tukey's test. Individual pairwise comparisons were made with the unpaired *t* test with Bonferroni's correction after ANOVA analysis. Urinary norepinephrine was significantly influenced by strain (*P*<0.01), azilsartan (*P*<0.01), and period (*P*<0.01). Urine aldosterone was significantly influenced by strain (*P*<0.01), azilsartan (*P*<0.01), and period (*P*<0.01). #*P*<0.05 *P<0.01 vs V within the same strain and the same period. WKY and W indicate Wistar–Kyoto rats; S, SHR rats; SHRcp and cp, SHR/NDmcr‐cp(+/+) rats; SEM, standard error of the mean; ANOVA, analysis of variance; V, vehicle treatment; A, azilsartan treatment.

**Figure 7.**
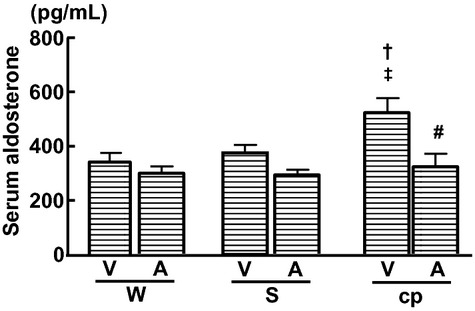
Effects of azilsartan on serum aldosterone levels in WKY, SHR, and SHRcp. Values are the mean±SEM (n=6 in each group). Statistical analysis was performed by 2‐factor ANOVA followed by a post hoc Tukey's test. Serum aldosterone was significantly influenced by strain (*P*<0.01) and azilsartan (*P*<0.05). #*P*<0.05 vs V within the same strain; ‡*P*<0.05 vs vehicle group of WKY rats; †*P*<0.05 vs vehicle group of SHR rats. WKY and W indicates Wistar–Kyoto rats; SHRcp, SHR/NDmcr‐cp(+/+) rats; SEM, standard error of the mean; ANOVA, analysis of variance; V, vehicle treatment; A, azilsartan treatment.

SHRcp exhibited greater urinary norepinephrine (*P*<0.01) and aldosterone (*P*<0.01) excretion than age‐matched WKY and SHR during dark and light periods (Figure 6). As shown in Figure 6, azilsartan treatment significantly reduced urinary norepinephrine and urinary aldosterone excretion in SHRcp during the dark and light periods. On the other hand, azilsartan did not significantly alter urinary norepinephrine excretion and urinary aldosterone excretion in WKY. As shown in Figure 7, SHRcp showed higher serum aldosterone levels than WKY (*P*<0.05) and SHR (*P*<0.05). Azilsartan significantly decreased serum aldosterone levels in SHRcp (*P*<0.05).

**Figure 8.**
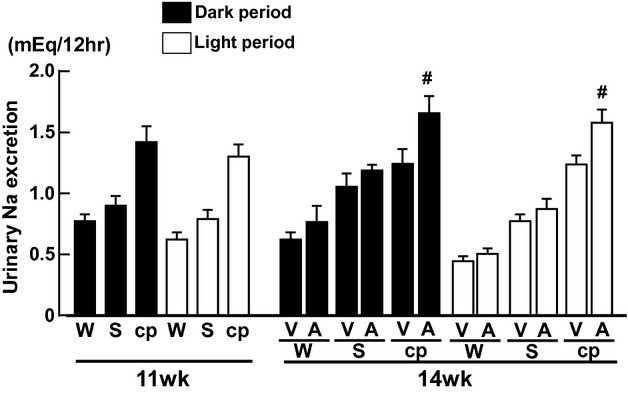
Effects of azilsartan on urinary sodium excretion in WKY, SHR, and SHRcp over 24 hours (12‐hour dark and 12‐hour light periods). Values are the mean±SEM (n=12 in each group of 11‐week‐old rats, n=6 in each group of 14‐week‐old rats). Statistical analysis was performed by the Kruskal–Wallis test followed by a post hoc Steel–Dwass's multiple comparison test. #*P*<0.05 vs V within the same strain and the same period. WKY and W indicate Wistar–Kyoto rats; S, SHR rats; SHRcp and cp, SHR/NDmcr‐cp(+/+) rats; SEM, standard error of the mean; V, vehicle treatment; A, azilsartan treatment.

Figure 8 shows that azilsartan treatment for 3 weeks (at 0.1 mg/kg for the first week, 0.3 mg/kg for the second week, and 1 mg/kg for the third week) significantly increased urinary sodium excretion in SHRcp during the dark and light periods, whereas azilsartan did not affect them in WKY and SHR.

### Circadian Rhythms of Autonomic Function in SHRcp and the Effect of Azilsartan

**Figure 9.**
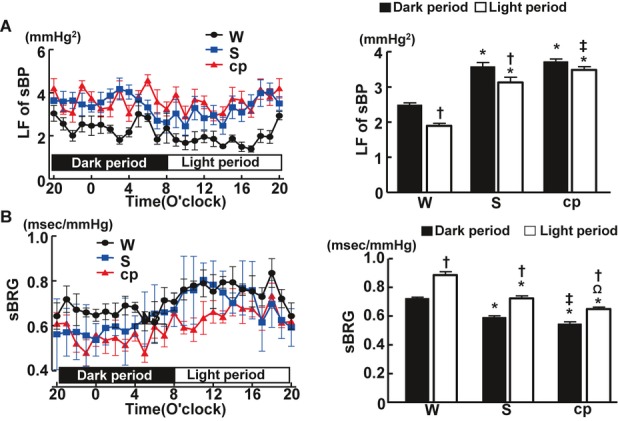
Circadian rhythms of low‐frequency power (LF) of systolic blood pressure (sBP) (A) and spontaneous baroreceptor reflex gain (sBRG) (B) in WKY, SHR, and SHRcp revealed by 24‐hour recording. Left panel in (A) indicates hourly recordings of LF of sBP over 24 hours, and right panel in (A) indicate the average LF of systolic BP during 12‐hour dark and 12‐hour light periods. Left panel in (B) indicates hourly recordings of sBRG over 24 hours, and right panel in (B) indicate the average sBRG during 12‐hour dark and 12‐hour light periods. Values are the mean±SEM (n=5 in each group). Statistical analysis was performed by 2‐factor ANOVA followed by a post hoc Tukey's test. LF of sBP was significantly influenced by strain (*P*<0.01) and period (*P*<0.01). sBRG was significantly influenced by strain (*P*<0.01) and period (*P*<0.01). *P<0.01 vs W within the same period; †*P*<0.01 vs the same strain in the dark period; ‡*P*<0.05; Ω*P*<0.01 vs S within the same period. WKY and W indicate Wistar–Kyoto rats; S, SHR rats; SHRcp and cp, SHR/NDmcr‐cp(+/+) rats; SEM, standard error of the mean; ANOVA, analysis of variance.

As shown in Figure 9A, in both SHR and WKY, LF of systolic BP was lower during the light period than the dark period (*P*<0.01). On the other hand, LF of systolic BP in SHRcp was similar between the dark and light periods. LF of systolic BP in SHRcp was higher than that in WKY during the dark (*P*<0.01) and light (*P*<0.01) periods and was higher than that in SHR during the light period (*P*<0.05). As shown in Figure 9B, sBRG in SHRcp was lower than that in WKY during the dark (*P*<0.01) and light (*P*<0.01) periods and was also lower than that in SHR during the dark (*P*<0.05) and light (*P*<0.01) periods.

**Figure 10.**
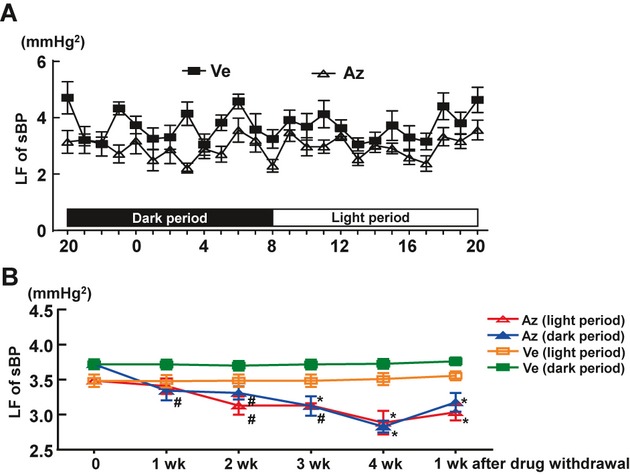
Effect of azilsartan on circadian rhythms of low frequency power (LF) of systolic blood pressure (sBP) in SHRcp revealed by 24‐hour recording. A, indicates hourly recordings of LF of systolic BP over 24 hours (during 12‐hour dark and 12‐hour light periods) in SHRcp after 4 weeks of azilsartan (1 mg/kg per day) or vehicle treatment. B, indicates the 12‐hour average LF of systolic BP during dark and light periods in SHRcp before (0) and 1, 2, 3, and 4 weeks after start of azilsartan (1 mg/kg per day) or vehicle treatment, and 1 week after azilsartan or vehicle withdrawal. Az, SHRcp treated with azilsartan (1.0 mg/kg per day); Ve, SHRcp treated with vehicle. Values are the mean±SEM (n=5 in each group). Statistical analysis was performed by 2‐factor ANOVA with repeated measures followed by post‐hoc Tukey's test. LF of sBP was significantly influenced by azilsartan (*P*<0.01) and period (*P*<0.01). #*P*<0.05 *P<0.01 vs vehicle within the same period at the same time point. SEM indicates standard error of the mean; ANOVA, analysis of variance.

**Figure 11.**
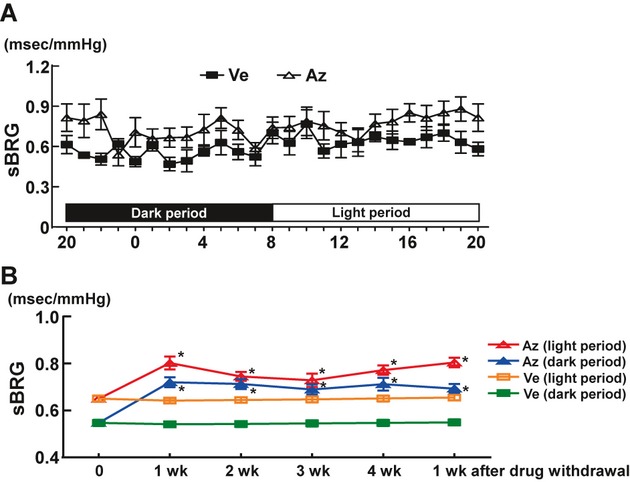
Effects of azilsartan on spontaneous baroreceptor reflex gain (sBRG) in SHRcp revealed by 24‐hour recording. A, Hourly recordings of sBRG over 24 hours (during dark and light periods) in SHRcp after 4 weeks of azilsartan (1 mg/kg per day) or vehicle treatment. B, The 12‐hour average sBRG in SHRcp before (0) and 1, 2, 3, and 4 weeks after start of azilsartan (1 mg/kg per day) or vehicle treatment and 1 week after azilsartan or vehicle withdrawal. Values are the mean±SEM (n=5 in each group). Statistical analysis was performed by 2‐factor ANOVA with repeated measures followed by a post hoc Tukey's test. sBRG was significantly influenced by azilsartan (*P*<0.01) and period (*P*<0.01). *P<0.01 vs vehicle within the same period at the same point. SHRcp indicates SHR/NDmcr‐cp(+/+) rats; SEM, standard error of the mean; ANOVA, analysis of variance.

**Figure 12.**
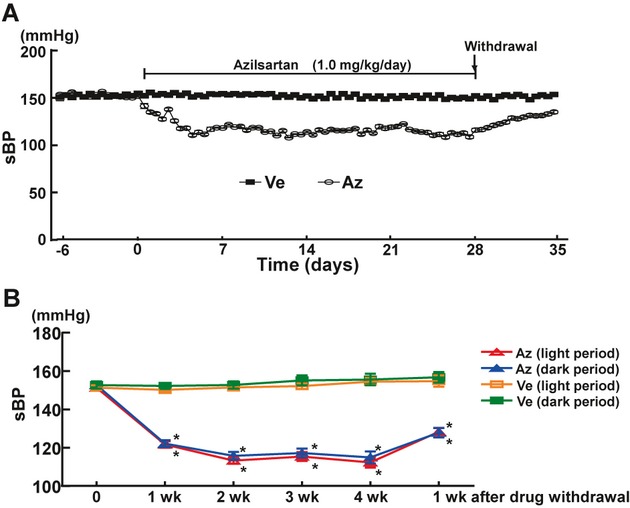
Effects of azilsartan on systolic blood pressure (sBP) of SHRcp during dark and light periods. A, The 12‐hour average sBP of SHRcp during dark and light periods. Azilsartan at 1.0 mg/kg or vehicle was given to SHRcp by gastric gavage once a day for 28 days. B, The 12‐hour average sBP of SHRcp during dark and light periods before (0) and 1, 2, 3, and 4 weeks after start of azilsartan or vehicle treatment and 1 week after azilsartan or vehicle withdrawal. Values are the mean±SEM (n=5 in each group). Statistical analysis was performed by 2‐factor ANOVA with repeated measures followed by a post hoc Tukey's test. sBP was significantly influenced by azilsartan (*P*<0.01) but not influenced by period (*P*=0.3035). *P<0.01 vs vehicle within the same period at the same time. SHRcp indicates SHR/NDmcr‐cp(+/+) rats; SEM, standard error of the mean; ANOVA, analysis of variance.

Compared with vehicle treatment, azilsartan treatment significantly reduced the LF of systolic BP (Figure 10) and significantly increased sBRG (Figure 11) in SHRcp during the dark and light periods. Moreover, compared with vehicle, the significant decrease in the LF of systolic BP and the significant increase in sBRG in SHRcp continued 1 week after azilsartan withdrawal (Figures 10 and 11). Compared with vehicle, the significant reduction in systolic BP in SHRcp also continued 1 week after azilsartan withdrawal (Figure 12).

### Effect of Short‐Term Azilsartan Treatment on Urinary Sodium Excretion in SHRcp

**Figure 13.**
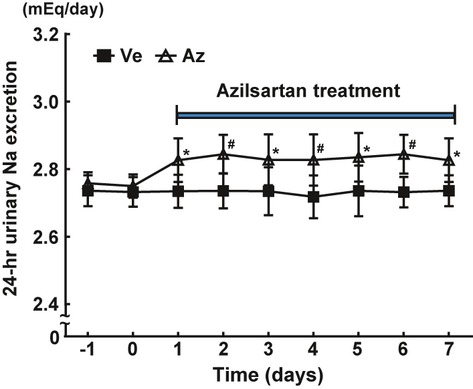
Effects of short‐term azilsartan treatment on 24‐hour urinary sodium excretion in SHRcp. Values are expressed as the mean±SEM (n=6 in each group). Statistical analysis was performed by 1‐factor ANOVA with repeated measures followed by a post hoc Bonferroni's multiple comparisons test. Urinary sodium excretion was significantly influenced by azilsartan (*P*<0.05). #*P*<0.05; *P<0.01 vs vehicle at the same time. SHRcp indicates SHR/NDmcr‐cp(+/+) rats; SEM, standard error of the mean; ANOVA, analysis of variance.

Figure 13 showed that short‐term oral azilsartan administration once daily significantly increased 24‐hour urinary sodium excretion compared with vehicle.

## Discussion

Impaired circadian BP rhythm is frequently observed in hypertensive patients with MetS^1–2^ and is significantly associated with an increased risk of cardiovascular events.^3–8^ However, investigation of the mechanism of disrupted circadian BP rhythms in MetS has been hampered by the lack of a suitable animal model. The major findings of this work are as follows: (1) SHRcp display nondipper hypertension, differing from SHR, and are therefore a suitable animal model for studying the mechanism underlying abnormal circadian BP rhythm in MetS; and (2) it is likely that this disrupted circadian BP rhythm in SHRcp was at least in part attributed to angiotensin II–mediated autonomic dysfunction and enhanced aldosterone levels. Therefore, our present work has provided novel insight into the mechanism of impaired diurnal BP variation in MetS.

Although SHRcp had much less locomotor activity than control WKY and SHR during the dark/active period, SHRcp had much higher BP than WKY over 24 hours (dark and light periods), as estimated by radiotelemetry. In contrast to SHRcp, obese Zucker rats with a mutation in the leptin receptor–encoding gene^23–25^ have very mild BP elevation compared with control lean rats, and leptin receptor‐deficient db/db mice^26^ have normal BP during the dark period and only slightly higher BP (by 9 mm Hg) during the light period than control mice. Therefore, our present study has demonstrated that SHRcp are a useful model for studying the mechanism of hypertension in MetS. However, further study is needed to determine whether the SHRcp rate is a more useful model to elucidate the mechanism of obese hypertension than other obese models.

In the present work, continuous BP monitoring using telemetry showed that SHR exhibited dipper‐type hypertension, which was in good agreement with previous reports.^27–29^ Of note, BP in SHRcp was comparable in the dark/active and light/inactive periods. Our present work has demonstrated that unlike SHR, SHRcp exhibited nondipper‐type hypertension, as is the case of hypertensive patients with MetS. Furthermore, we found that the difference in HR between dark and light periods in SHRcp was less than that in SHR or WKY. This unique HR circadian rhythm in SHRcp seems to be partially attributed to their having less locomotor activity than SHR or WKY, although future work is required to define the precise mechanism. To elucidate the potential role of sympathetic nerve activity in hypertension in SHRcp, we compared the LF of systolic BP and sBRG in SHRcp with that of WKY and SHR by radiotelemetry combined with spectral analysis using a fast Fourier transformation algorithm. Interestingly, the LF of systolic BP in SHRcp was greater than that in WKY and SHR, and sBRG in SHRcp was less than that in WKY and SHR over 24 hours. Furthermore, urinary norepinephrine excretion was greater in SHRcp than in WKY and SHR. These results demonstrate that SHRcp were characterized by an enhanced vasomotor sympathetic tone and impaired baroreceptor reflex function, thereby supporting the involvement of autonomic dysfunction in nondipper hypertension in SHRcp.

To examine the potential role of the renin‐angiotensin system in hypertension in SHRcp, we compared the effect of azilsartan,^14,30^ a new ARB, on SHRcp with that on SHR and WKY. An in vitro study^14^ has shown that azilsartan has a higher affinity for and slower dissociation from the AT1 receptor than other ARBs (olmesartan, telmisartan, valsartan, and irbesartan). Furthermore, recent clinical studies of Western^13,16–17^ and Japanese^15^ hypertensive patients have demonstrated that once‐daily azilsartan administration lowers 24‐hour BP persistently and improves nocturnal hypertension more effectively than other ARBs such as olmesartan, valsartan, and candesartan, suggesting that azilsartan may be a promising ARB for the treatment of hypertension with abnormal circadian rhythm. However, little is known about the potential mechanisms underlying azilsartan‐induced BP normalization. Therefore, in the present work, we used azilsartan as an ARB to elucidate the potential role of the renin‐angiotensin system in the mechanism of hypertension in SHRcp. Consistent with clinical data showing the long‐lasting BP‐lowering effect of azilsartan in hypertensive patients,^13,15–17^ once‐daily dosing of azilsartan in SHRcp produced persistent BP lowering over 24 hours, as shown by comparable BP lowering in the dark and light periods. Furthermore, it should be noted that the BP‐lowering effect of azilsartan was greater in SHRcp than in SHR and WKY, as shown by the greater reduction in MAP by various doses of azilsartan in SHRcp than in SHR and WKY. These results provided the evidence that the AT1 receptor plays a major role in disrupting the diurnal BP variation in SHRcp.

To determine the detailed mechanism responsible for persistent BP lowering by azilsartan in SHRcp, we examined the effect of azilsartan on the autonomic nervous system. We found that azilsartan significantly reduced the LF of systolic BP over 24 hours (dark and light periods) and significantly increased sBRG over 24 hours in SHRcp. Furthermore, azilsartan significantly decreased day and night urinary norepinephrine excretions in SHRcp compared with WKY and SHR. These observations suggest that greater BP reduction by azilsartan over 24 hours in SHRcp rats than in WKY and SHR rats was attributed, at least in part, to amelioration of vascular sympathetic activity and improvement in baroreceptor reflex function.

In addition to having an impaired autonomic nervous system, we found that, unlike SHR, SHRcp were characterized by an increase in circulating aldosterone, which is consistent with previous studies.^10–11^ Of note, azilsartan significantly reduced urinary aldosterone excretion during the dark and light periods and decreased plasma aldosterone in SHRcp, in contrast to no change in aldosterone by azilsartan in WKY and SHR. Moreover, azilsartan significantly increased urinary sodium excretion in SHRcp but not in WKY and SHR. Collectively, our present findings suggest that greater long‐lasting BP reduction induced by azilsartan in SHRcp is partially attributed to a reduction in aldosterone. However, further study is needed to elucidate the possible contribution of natriuresis by azilsartan to blood pressure lowering, because it cannot be ruled out that the increase in sodium excretion by azilsartan in the chronic stage might reflect sodium intake.

In conclusion, SHRcp rats are a valuable model for studying the mechanism of circadian BP rhythm disorder in MetS. The mechanism of hypertension in this new rat model of MetS was attributed to enhanced vascular sympathetic nerve activity, impaired baroreceptor function, and increased aldosterone levels. Our results strongly suggest that impairment of autonomic function and increased aldosterone in SHRcp mediate the effect of angiotensin II on circadian blood pressure rhythms. Furthermore, the present findings provide a novel and useful experimental rationale for the treatment of hypertension with azilsartan. However, further study is warranted to define the potential role of obesity itself in disrupted BP circadian rhythm in SHRcp. Furthermore, as a potential study limitation, it cannot be excluded that several of the comparisons might be underpowered because of the small sample size in each group.

### Perspectives

MetS is closely associated with the pathogenesis of hypertension and increases the risk of cardiovascular events. Most hypertensive patients with MetS are characterized by a disrupted circadian BP rhythm, such as nondipper‐, riser‐, and morning surge–type hypertension. Such disrupted circadian BP rhythms significantly increase the risk of cardiovascular events. The precise mechanism of circadian BP rhythm disorder in MetS remains to be defined because of a lack of a suitable experimental animal model. In the present work, we have demonstrated that SHRcp exhibit nondipper‐type hypertension and are a useful model for studying the mechanism of abnormal circadian BP rhythm in MetS. We also suggest that angiotensin II–mediated abnormal regulation of the autonomic nervous system and aldosterone are responsible for the disrupted circadian BP rhythm in SHRcp. Therefore, our present work not only provides a novel finding on the mechanism of disrupted circadian BP rhythm in MetS but also highlights the autonomic nervous system and renin‐angiotensin‐aldosterone system as promising therapeutic targets for hypertension complicated by MetS. Furthermore, our present work shows that azilsartan, a new ARB, is a promising agent for the treatment of hypertension with disrupted circadian rhythm.
